# Father Absence and Reproduction-Related Outcomes in Malaysia, a Transitional Fertility Population

**DOI:** 10.1007/s12110-014-9195-2

**Published:** 2014-03-08

**Authors:** Paula Sheppard, Kristin Snopkowski, Rebecca Sear

**Affiliations:** Department of Population Health, London School of Hygiene and Tropical Medicine, Keppel Street, London, WC1E 7HT UK

**Keywords:** Father absence, Life history strategy, Menarche, Reproduction, Demographic transition

## Abstract

Father absence is consistently associated with children’s reproductive outcomes in industrialized countries. It has been suggested that father absence acts as a cue to particular environmental conditions that influence life history strategies. Much less is known, however, about the effects of father absence on such outcomes in lower-income countries. Using data from the 1988 Malaysian Family Life Survey (*n* = 567), we tested the effect of father absence on daughters’ age at menarche, first marriage, and first birth; parity progression rates; and desired completed family size in Malaysia, a country undergoing an economic and fertility transition. Father absence during later childhood (ages 8 to 15), although not during earlier childhood, was associated with earlier progressions to first marriage and first birth, after controlling for other confounders. Father absence does not affect age at menarche, desired family size, or progression from first to second birth. The patterns found in this transitional population partly mirror those in developed societies, where father absence accelerates reproductive events. There is, however, a notable contrast between the acceleration in menarche for father-absent girls consistently found in developed societies and the lack of any association in our findings. The mechanisms through which father absence affects reproduction may differ in different ecological contexts. In lower-income contexts, direct paternal investment or influence may be of more importance in determining reproductive behavior than whether fathers act as a cue to environmental conditions.

**Electronic supplementary material:**

The online version of this article (doi:10.1007/s12110-014-9195-2) contains supplementary material, which is available to authorized users.

A large body of research has emerged that examines the relationship between father absence and the timing of children’s life transitions and reproductive events. However, the great majority of these studies have been conducted in low-fertility, post–demographic transition settings. In higher fertility societies, both pre–demographic transition and transitional societies (where fertility rates are declining but have not reached replacement levels), researchers have looked at the effects of fathers on infant and child mortality, along with other health outcomes (Sear and Coall 2011; Sear and Mace 2008; Sear et al. 2002; Winking et al. 2009), but much less is known about the relationship between father absence in childhood and demographic outcomes in later life, such as fertility. Pre-demographic transition and transitioning societies differ in a number of ways (for example, differing divorce rates, marital patterns, postnuptial residence, and rates of higher education) from post–demographic transition societies, which may influence the relationship between father absence and life history events. Henrich et al. (2010) have argued that high-income, post-demographic transition populations constitute a particularly “WEIRD” group—Western, educated, industrialized, rich, and democratic, which does not characterize most of the world. It is therefore important to test predictions that hold up in WEIRD settings in ones that more accurately represent the majority of the world’s societies. This study aims to contribute to current understanding of the relationship between father absence and reproduction-related outcomes by extending such knowledge to a less WEIRD population.

In this study we test whether father absence is related to age at menarche and other fertility-related outcomes (age at marriage, age at first birth, progression to births, and desired fertility) in a transitional setting. Most research focuses on only one or two of these elements, and few empirically examine both physiological (puberty) and behavioral (e.g., age at marriage) outcomes in the same study population. As a result, there is no clear interpretation of how father absence influences both kinds of outcomes. The assumption underlying such studies is often that any one of these traits can stand as a good proxy for another, and that these events form part of a suite of interrelated characteristics leading to reproduction. This is perfectly plausible, and obviously the ability to reproduce early has to be preceded by other factors such as puberty and sex in order to accomplish that goal, but here we explicitly test whether father absence has the same effect on both physiological and behavioral outcomes. Below, we outline the different theories given for the relationship between father absence and our main outcomes.

## Father Absence and Age at Puberty

In low-fertility, post–demographic transition settings it has been consistently found that father absence (or low paternal investment) is associated with earlier age at puberty, at least in women (Alvergne et al. 2008; Bogaert 2008; Ellis et al. 1999, 2012; Hoier 2003; Maestripieri et al. 2004; Moffitt et al. 1992; although see Kiernan and Hobcraft 1997). Two adaptive hypotheses have been proposed for this relationship (for detailed reviews on the father absence-puberty literature see Ellis 2004 and Belsky 2012). First, early psychosocial stress, including father absence, has been suggested to be responsible for accelerating age at menarche (Belsky et al. 1991; Ellis 2004; Moffitt et al. 1992). Chisholm et al. (2005) expand this explanation and argue that psychosocial stress is a cue to a shortened lifespan that leads to accelerated ages at menarche and earlier reproduction where the risk of dying before producing significant numbers of offspring is high. Both models imply that father absence is but one type of childhood psychosocial stress and that there is nothing about the lack of a father per se that, by itself, affects reproductive events. These arguments also suggest that early life conditions drive the propensity to start reproducing earlier: age at menarche is reduced under stressful early life conditions because such conditions promote an overall fast life-history strategy.

As far as we are aware no one has yet looked at the relationship between father absence and pubertal timing among hunter-gatherer or other nutritionally stressed societies. However, we know that age at menarche is associated with health and nutrition as a number of studies show that poor nutrition delays pubertal maturation in girls (Khan et al. 1996; Simondon et al. 1998; Walker et al. 2006) and in boys (Kuzawa et al. 2010; Walker et al. 2006). In an environment of poor nutrition, it is better to grow more slowly, for longer, and remain smaller as an adult, thereby incurring fewer metabolic expenses later on (Ellison 2001). This ultimately delays puberty and reproduction. Therefore, absent fathers may not consistently be associated with accelerated life histories in all contexts because father absence may equate to nutritional stress (in cases where fathers provision children or provide other forms of resources) (Ellis 2004). However, the role of fathers varies considerably cross-culturally, so their absence may result in varying degrees of nutritional and/or psychosocial stress. To be able to predict father absence effects on menarche in a particular population, both the level of resource access and the role of the father need to be taken into account.

## Father Absence and Reproduction-Related Behavior

Here we refer to age at marriage, age at first birth, and birth spacing as behavioral traits because we are interested in reproductive behavior as well as the physiological antecedents to such behavior. We also consider ideal family size as a rough proxy for actual competed fertility (since we do not have data on actual completed fertility for the women in the sample). Father absence in a post–demographic transition context is found to be associated with earlier first sexual experience (Matchock and Susman 2006; Quinlan 2003), and earlier first births in both women (Ellis et al. 2003; Nettle et al. 2010) and men (Kiernan 1992; Sheppard and Sear 2012). Similar arguments to those proposed for menarche have also been put forward for reproduction-related behavior—in other words, that father absence during early childhood is a single component of early life stress which results in an overall faster reproductive trajectory (Belsky et al. 1991). From an adaptive point of view (all else being equal), in environments where life expectancy is low or highly variable and thus unpredictable, it is preferable to start reproduction earlier rather than later in order to maximize one’s reproductive span, offset the possibility of premature death, and shorten generational length to maximize lineage descendants (Chisholm 1993). Some empirical evidence from contemporary Australia supports this prediction: women who experienced early childhood stress reported shorter expected lifespans and also had earlier first births compared with women who reported having low-stress childhoods (Chisholm et al. 2005). An alternative hypothesis, and in fact the earliest proposed adaptive hypothesis for behavioral differences between father-absent and father-present girls, is that growing up in a father-absent environment indicates that marriages are unstable and that men are unreliable investors (Draper and Harpending 1982; Ellis and Essex 2007; Ellis et al. 2003; Geary 2000). In such environments it is advantageous for the daughter to reproduce younger and not delay reproduction while waiting for an ideal, highly investing mate when that prospect is unlikely to happen.

Few empirical studies have examined the relationship between father absence and fertility outcomes in pre–demographic transition societies. Those that have report mixed results. Among Ache foragers and Tsimane forager-agriculturalists, the death of a father had no impact on fertility rates or age at first birth, respectively, for either sons or daughters (Hill and Hurtado 1996; Winking et al. 2011). In another study however, Waynforth et al. (1998) found that father absence due to divorce was associated with delayed age at reproduction, for both Belizean Maya agriculturalists and Paraguayan Ache forager males. This was possibly because sons who received reduced paternal investment had difficulty acquiring mates. Martu Aboriginal Australian fathers help sons by arranging an initiation ritual which enables them to marry and reproduce sooner than fatherless boys: although substituted (usually related) father figures also buffered against later initiations, the strongest effect was nevertheless from biological fathers (Scelza 2010). For women, a weak relationship was also observed between father absence and delayed first births in Gambian agriculturalists (Allal et al. 2004), again perhaps because of the importance of fathers for arranging marriages in this population.

Among historical Finns, father presence was found to be associated with younger ages at first birth as well as shortened interbirth intervals, leading to lengthened reproductive life spans in firstborn sons, but not later born sons or daughters. Exactly why the presence of fathers was correlated with longer reproductive tenure in their firstborn sons is not clear, but the authors suggest it may be related to primogeniture land inheritance patterns in this population (Lahdenperä et al. 2007). Furthermore, two studies investigate a population perhaps most similar in fertility rate to the transitional population studied here. Flinn (1986) found that having a resident father increased young adult male, but not female, reproductive success (defined as offspring surviving for more than one month) in a rural Trinidadian village. In this population, fathers were also found to engage in “daughter-guarding” behaviors, likely to reduce the sexual activity of teenage girls (Flinn 1988). This was suggested as an attempt to manage daughters’ reputations and improve their chances of entering into a prosperous marriage, though whether this behavior influenced daughters’ age at marriage or at first birth was not tested (Flinn 1988). In rural Bangladesh, Shenk et al. (2013) tested the effects of different types of father absence: divorce/deserted, death, and migrant work (making these fathers at least partly absent). They found that father-absence effects are not consistent across type of absence. Daughters of divorced fathers had earlier ages of marriage and first birth, whereas daughters of dead or migrant fathers had later ages of marriage and first birth compared with father-present women. These results suggest that it is the type of father absence that is a more important influence on Bangladeshi girls’ reproductive strategies than absence per se.

The above examples indicate that a fourth hypothesis may explain the link between father absence and reproductive outcomes: instead of being a cue to a particular set of environmental conditions, paternal investment may directly influence entry into marriage and parenthood. This may be either because fathers are important as providers of material resources, which will influence nutrition and growth in a resource-poor setting, or because fathers are important as facilitators of social rites or monitors of sexual behavior which affect the timing of entry into sexual behavior and marriage. Note that under this hypothesis the predicted direction of the father effect is context-dependent and is not consistent. For example, if fathers guard daughters, then father absence should lead to earlier marriage and first births; however, if they are required to arrange a marriage, then father absence may delay these events. Finally, we may expect different effects depending on the sex of the child. In a situation where fathers provide financial resources, father absence may delay a son’s ability to acquire a wife but a daughter may marry younger in order to attain financial security by entering into a marriage of her own.

In summary, four hypotheses have been proposed to explain the link between fathers and reproduction-related behavior: (1) father absence may be an indicator of a stressed early environment (Belsky et al. 1991), (2) father absence may be a mortality cue (Chisholm 1993), which results in an accelerated life history strategy; (3) father absence may indicate the degree to which a woman can expect paternal investment in her future reproductive career (Draper and Harpending 1982); (4) paternal investment may directly influence the reproductive behavior of their offspring (Allal et al. 2004; Flinn 1988; Scelza 2010). In an attempt to distinguish between these hypotheses, we not only nave investigated the effects of father absence on several different reproductive outcomes in our data but have also attempted to determine whether fathers are uniquely important, whether the timing of their absence matters, and whether the cause of absence matters.

## Are Fathers Special?

Overwhelming evidence shows that early life stress can accelerate the timing of later reproduction-related outcomes in a developed world setting (Brumbach et al. 2009; Chisholm et al. 2005; Coall and Chisholm 2010; James-Todd et al. 2010; Kuzawa et al. 2010; Nettle 2010). The first two hypotheses above consider father absence to be a psychosocial stressor within an overall stressed rearing environment; the second two hypotheses suggest that there is something special about fathers and so they should influence their offspring’s reproductive outcomes over and above other early life stress. In order to test these alternative models, it is important to isolate father effects from other early stressors, wherever possible. Belsky et al.’s (1991) assertion that psychosocial stress is the important factor is also an assumption that needs testing. It is not easy to pick apart resource stress from psychosocial stress because poverty could well lead to psychosocial stress, making it difficult to know which mechanism may be driving the father absence effect. Some studies that have controlled for other early life stressors suggest there may indeed be something intrinsic about fathers that matters since father absence remains associated with their children’s reproductive outcomes net of these controls (Ellis et al. 2003; Maestripieri et al. 2004; Quinlan 2003; Sheppard and Sear 2012). We were able, in the current study, to alleviate some of these confounders by adjusting for parental household wealth in our models, although these data do not allow us a more nuanced treatment of childhood stress because we have no information on other types of non-resource-related stressors during childhood.

## Does the Timing of Father Absence Matter?

Previous research has suggested that early childhood, usually before age five or seven, is the crucial period in which father absence has consequences for later outcomes (Belsky et al. 1991; Chisholm et al. 2005; Draper and Harpending 1982). Recently this line of reasoning has been challenged, and empirical research points to a more complex relationship. It seems that father absence is important at different childhood stages in different ways. For example, Alvergne et al. (2008) found that, in contemporary France, father absence before age five was associated with earlier menarche, but it was father absence during adolescence that correlated with heightened sexual activity. Using data from urban India, Shenk and Scelza (2012) found that the death of one’s father during later childhood or adolescence was associated with marrying poorer quality mates, not the loss of fathers early in childhood. In another study, Sheppard and Sear (2012) found that father absence before age seven was associated with earlier siring of children in British men but that father absence during adolescence was related to delayed voice breaking (an indicator of puberty). Quinlan (2003) found that, among American women, father absence before age five was associated with earlier menarche, first sexual intercourse, and pregnancy, although father absence occurring during adolescence was associated with sexual promiscuity. This may be because fathers influence their daughters’ reproduction through different mechanisms, and these mechanisms require father absence at different developmental stages. Although the three hypotheses described above that consider father absence to be a cue to a particular environment may require fathers to be absent during a “sensitive period” in early childhood, father absence during later childhood or adolescence may be more important for the fourth hypothesis, which suggests that paternal behavior directly influences the ability to begin reproductive behavior. Testing for father absence effects at different childhood stages may therefore enable us to gain an insight into which mechanism (or mechanisms) is important in a particular population.

## Causes of Father Absence

Previous research, in high-income countries, has shown that parental divorce tends to result in more detrimental outcomes for children than the death of a father (Biblarz and Gottainer 2000; Kiernan 1992). This is sometimes attributed to the likelihood that the familial conditions that lead up to divorce create an extended period of conflict and stress in the home whereas death usually does not (Amato 1993). There may also be something systematically different about parents who divorce compared with those who don’t, whereas death can be seen as a relatively random occurrence. Biblarz and Gottainer (2000) report that divorced and never-married mothers have significantly lower-status occupations, earn substantially less, and have lower levels of job satisfaction than widows do. Widows may therefore be able to invest more in their children than divorced or never-married women. An alternative explanation for correlations between family structure and reproductive events is that some women have genetic predispositions toward a certain life history strategy (e.g., one that links early puberty and reproduction with unstable partnerships), and that their daughters inherit these predispositions (Campbell and Udry 1995). These factors may all contribute to a more difficult childhood for children from divorced parents compared with those who suffered the death of their father.

Studies conducted among pre-transitional populations tend not to compare the effects of death and divorce directly; usually only one type of father absence is considered with regard to children’s outcomes (perhaps because sample sizes tend to be smaller when pre-transitional populations are studied), although for a recent exception, see Shenk et al. (2013). It is therefore tricky to make any empirically based predictions about what we would expect in such settings. Theoretically, however, the first hypothesis (above) suggests that father absence through either death or divorce should cause psychosocial stress, so that cause of father absence should matter little; the second hypothesis, that father absence is a mortality cue, would imply that father death would be more important than parental divorce; the third hypothesis involves father absence as a cue to the population’s mating strategy, so that divorce should matter but not paternal death. Finally, if fathers are necessary for practical reasons, as in hypothesis 4, we would expect death to have a more detrimental effect, assuming that divorced fathers may continue to invest in their children to some extent, unless divorce brings about a large geographic separation from children and in effect constitutes the permanent loss of the father. The data we use here allow us to compare father absence due to death and divorce to see if any differences in cause of father absence are discernible in such a population.

The current study aims to examine the relationship between father absence and later fertility outcomes in a transitional context; Malaysia. This study captures data during a period of rapid decline in fertility and mortality; the data were collected from women born between 1940 and 1971. If father absence influences nutritional stress in a natural fertility population, and psychosocial stress in a low-fertility context, where would an intermediate society fit in? Using data from a transitional population to examine father effects on fertility enables us to broaden our knowledge on reproductive strategies.

## Data

We use data collected by the RAND Corporation in Malaysia (RAND 2012). The Malaysian Family Life Surveys (MFLS) consists of an initial study conducted in 1976–1977 on a representative sample of all ever-married women under the age of 50, randomly sampled from Peninsular Malaysia (i.e., excluding Sarawak and Sabah, the two provinces on the island of Borneo). Information was gathered on each focal woman, her husband, and other household members on retrospective and current information on a range of demographic topics, including family structure, household economics, and fertility (*N* = 1,262 households). A follow-up survey was administered in 1988–1989 in which 926 of the original households were reinterviewed (the “Panel sample”) as well as another sample of up to three adult children (over age 18) of the focal women (the “Children sample”). This study design allows us to look at long-term effects of father absence in relation to fertility decisions of adult children. Once we linked the focal women with their daughters we were left with a sample size of 567 adult daughters, which forms our sample for subsequent analysis.

Malaysia has undergone considerable economic growth over the past 60 years (for example, the human development index has increased from 0.563 in 1980 to 0.769 in 2012; United Nations 2013). Fertility rates have also fallen considerably during this period, with the 2009 total fertility rate (TFR) at 2.67 (The World Bank 2012). During the study period fertility was declining rapidly but had not yet reached this all-time low. The TFR dropped from 6.31 in 1960 to 3.58 in 1988 (reliable data are not available before 1960) (The World Bank 2012). Similarly, death rates have also dropped, with the child (under five) mortality rate dropping from 97 deaths per thousand children in 1960 to 20 deaths in 1988 (again, reliable data are unavailable for the decades preceding 1960) (The World Bank 2012).

Previous publications using MFLS data provide rich insight into the demographic trends of this cohort (Brien and Lillard 1994; Govindasamy and DaVanzo 1992; Leete 1989). Besides the fertility and mortality decline over this period, the demographic differences between ethnic groups are also notable. Malaysia has three primary ethnic groups: Malays (~65%), Chinese (~21%), and Indian (~14%) (Leete 1989). In Malaysia, Malays are predominantly matrilocal (i.e., residing with or nearby the bride’s family after marriage) whereas the Chinese and Indian groups are mostly patrilocal (i.e., postmarital residence with the husband’s family) (Smith and Thomas 1997).

Fertility has declined in all three groups, however, Malay women have had a much slower decline, and fertility plateaued in this group toward the end of this period. In 1988 the Chinese TFR was 2.3; the Indian TFR, 2.8; and Malay TFR, 4.5. This is partly explained by differing patterns of contraceptive use between the three groups; during the mid-1980s around 65% of Chinese and Indian women used contraception compared with only 41% of Malay women, and while this percentage had increased in the former two ethnic groups, it had become less popular for Malay, predominantly Muslim, women who, during the mid-seventies, went from using “the pill” as a favored choice of contraception to reverting to more traditional methods (Leete 1989).

Divorce rates also differ cross-culturally in Malaysia: from marriages commencing prior to 1968, Malay women (22.3%) experienced much higher levels of divorce than both Indian (5.7%) and Chinese (5.7%) women (Khalipah Mohd 1993). In the MFLS sample, 78.7% of divorced Malay women married again whereas less than half of Indian women remarried (42.8%) and only 19.9% of Chinese women did so. It is likely that divorce is more socially acceptable among Malay women and that their religious affiliation and cultural norms allow Malay women to remarry, while this is more difficult for Chinese or Indian women owing to their differing cultural backgrounds (Khalipah Mohd 1993).

## Methods and Models

We performed a multivariate linear regression analysis to determine the correlation between father absence and (1) age at puberty and a Poisson regression model for (2) desired family size. To examine the relationship between father absence and (3) progression to marriage, (4) progression to first birth, (5) progression from marriage to first birth, and (6) progression from first to second birth, we carried out discrete-time event history analyses in order to accommodate censored cases. We modeled each outcome of interest in three ways: father absence at any age before 16 (model A), father absence split into childhood age groups (model B), and father absence divided into causes of absence (model C). Descriptive statistics for outcome variables are shown in Table 1.

**Table 1:** Descriptive statistics for primary variables

Variable	n	Median	Min	Max
Age at menarche (yrs)	343	13	10	18
Desired family size	208	2	1	8
Age at marriage (yrs)^a^	548	20	13	30
Age at first birth (yrs)^a^	548	21	15	30
Length of first birth interval (marriage to first birth: yrs)^a^	328	1	1	5
Length of second birth interval (first to second birth: yrs)^a^	287	2	1	5
Wealth score	566	3	0	5
Family size	567	8	0	17

^a^Medians are calculated from both censored and uncensored cases

### Father-Absence Variables

Father absence was derived using the mothers’ marital histories and the year of birth of the daughter, allowing us to calculate the daughter’s age when father absence occurred. This variable was coded first as father absence occurring at any age from birth to age fifteen (*n* = 64), but then also into two groups—from birth to age seven (*n* = 32) and from age eight to fifteen inclusive (*n* = 32)—in order to investigate any timing effects. The reference category consists of 497 girls who experienced no father absence before age sixteen. Age seven was chosen as the dividing point because it is often proposed that experiences during early childhood (before ages five to seven) are especially important for affecting later life outcomes (Belsky et al. 1991; Chisholm et al. 2005; Draper and Harpending 1982). Studies looking at pubertal maturation in girls find age seven to be an important marker of adrenarche, a precursor to menarche (Ellis and Essex 2007). Re-running the models with age five or age ten as the cutoff did not significantly change the results. Finally, we derived a categorical variable for cause of father absence consisting of marital dissolution (divorce and separation combined, *n* = 22) and death (*n* = 43), with father presence as the reference category.

### Outcome Variables

#### Age at Menarche

Although both married and unmarried women were included in the survey, only married women were interviewed about their age (in years) at menarche (the measurement we use for puberty). This introduces a sample bias toward women who were married relatively young, which may be problematic because some evidence suggests that early age at menarche is correlated with marrying young (Borgerhoff Mulder 1989). We therefore limit our menarche models to women who were aged 25 or over at the time of the interview since 90% of these women were married by age 25. We included mother’s age at menarche in the model to control for genetic heritability. Three father-absent girls had already begun menarche before the father left. These girls were shifted to the father-present category and so we included only those girls who had lost their father before they started menses in the father-absence category.

#### Age at Marriage and First Birth

Using a discrete-time event history model to analyze progression to first marriage allows us to include censored women (there were 224 never-married adult women at the time of interview). The hazard of marriage was analyzed between the ages of 12 and 30 because 99% of those women who did marry had done so by age 30. In this model women thus become “at risk” for marriage at age 12. We know that marriage is the main predictor of age at first birth in this population. Nevertheless we also performed a discrete-time event history analysis on progression to first birth, limiting this analysis to include only women who had a first birth between age 15 and 30, and so women are at risk of a first birth from age 15. This model includes 270 censored women who had not yet had a first birth. We modelled both progression to marriage and first birth because in post–demographic transition populations, where marriage is a less important factor for predicting first birth, age at first birth is a commonly examined outcome. This way, we are able to make a more direct comparison with previous studies.

#### Birth Spacing

We also tested whether father absence had an effect on the pace of reproduction. We looked at progression from marriage to first birth and first to second births. A discrepancy has been shown between patterns of progression to first birth and subsequent ones: often, women have a first birth soon after marriage and then change tempo for later births (Bumpass et al. 1978; Eltigani 2000). We only considered first births that occurred within the first 5 years of marriage because births later than this in a pro-natalist society may result from fecundity problems. This only excluded four women (1%) from our sample.

#### Desired Completed Family Size

Finally, we examined whether father absence was associated with desired number of children. This is intended as a proxy for completed family size (not all woman in our sample had completed reproduction). Although this is far from a perfect measure, some evidence has shown that desired family size is a fair indicator of actual completed fertility (Bankole and Westoff 1998; DeSilva 1991; Freedman et al. 1975, but see Westoff et al. 1957). Furthermore, using data from both waves of the MFLS, DaVanzo et al. (2003) found that parents who had expressed a desire for more children went on to do so. Because this question was only asked of married women, we again included only women aged 25 or over in the models.

### Other Covariates

#### Wealth

Wealth has been shown to be associated with the timing of reproduction-related events, at least within a high income context (Geronimus 1996; Surbey 1998), so we included a measure of wealth in our models. It is also possible that any father-absence effects are mediated by wealth—in other words, that any father-absence effects observed are driven by the loss of resources a father can provide. A wealth variable was derived by adding up the total number of household possessions each woman had listed from her parental household: refrigerator, bicycle, car, television, and telephone. We treat this as a continuous variable in the model. Since we only had data available for the current parental household, using this variable assumes that parental household wealth is stable over time. Although we are not able to validate this assumption using these data, evidence among the Kenyan Kipsigis has shown that current parental wealth was a reliable measure of the household wealth in which daughters grew up (Borgerhoff Mulder 1989).

#### Total Family Size

We included variables for total family size (number of siblings of the respondent). Different parity children may be able to access household resources differentially, and given that previous research suggests that birth order may influence age at menarche (Matchock and Susman 2006), we also included this, as well as a quadratic term for birth order, in the model.

#### Ethnicity

Since there are systematic differences in fertility across ethnic groups, we control for ethnicity in all models. Ethnicity was coded as a binary variable—Malay and non-Malay (Chinese, Indian, and “other” combined)—because of the small sample sizes from the non-Malay ethnic groups, and because the Indian and Chinese groups follow similar patterns of fertility decline compared with the Malay women.

## Results

**Fig. 1:**
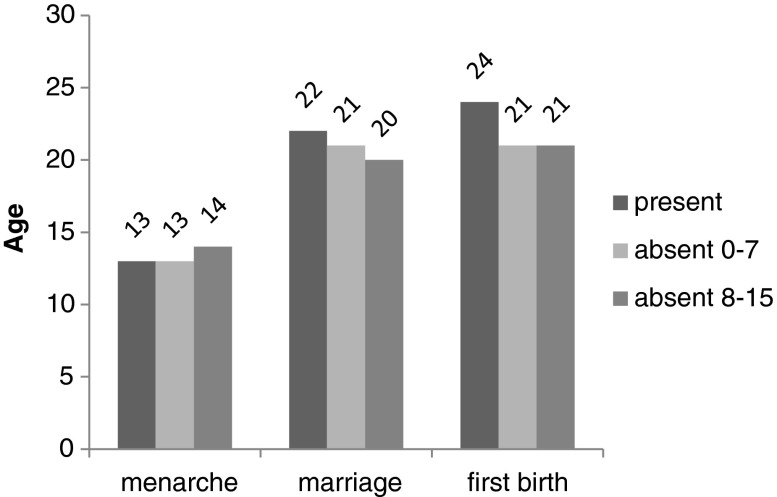
Median ages of major life history events, split into father absence categories

**Fig. 2:**
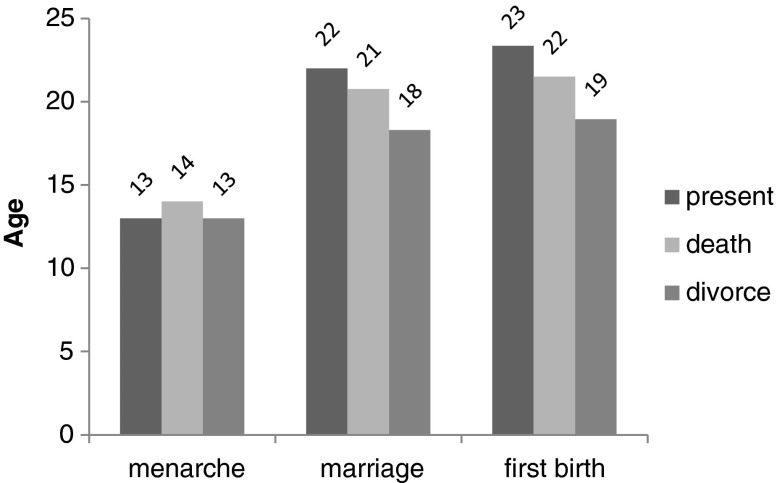
Median ages of major life history events, split into father absence causes

Figures 1 and 2 show the median ages of menarche, marriage, and first birth for father presence and absence, split into the childhood age-groups when the father became absent (Fig. 1) and the reason for his absence (Fig. 2). These figures, based on raw data, suggest that individuals who experienced father absence had earlier median ages of marriage and first birth but not menarche.

Results for all models are displayed in Table 2. Model A examines father absence at any stage during childhood, model B splits father absence into different age categories (early vs. later childhood), and model C tests different causes of father absence (death vs. divorce). All models adjust for wealth, ethnicity, total family size (number of siblings), birth order, and birth order squared. Menarche models further control for mother’s age at menarche. The event history analyses also adjust for age and age squared. We only present the variables of primary interest to this paper, but full tables of all models are available in the ESM. Event history models make the assumption that hazards are proportional over time. We satisfied this assumption in all such models as we found no significant interactions between each outcome of interest and time. Beta coefficients are displayed for the age at menarche and desired family size models; the event history analysis models report odds ratios.

**Table 2:** Results for all models

	Model A			Model B			Model C		
	β Coeff.	95% CI		β Coeff.	95% CI		β Coeff.	95% CI	
1. Age at puberty (*n* = 249)									
Father absent <16	−0.30	−0.85	0.25						
Father absent 0–7				−0.56	−1.25	0.14			
Father absent 8–15				0.12	−0.76	0.99			
Death							0.02	−0.74	0.79
Divorce							−0.63	−1.40	0.14
Wealth	−0.07	−0.21	0.06	−0.07	−0.21	0.06	−0.08	−0.21	0.06
Family size	0.10**	0.04	0.17	0.10**	0.04	0.17	0.11**	0.04	0.17
2. Ideal family size (*n* = 141)									
Father absent <16	0.05	−0.30	0.40						
Father absent 0–7				−0.01	−0.48	0.45			
Father absent 8–15				0.13	−0.39	0.65			
Death							0.04	−0.45	0.54
Divorce							0.06	−0.43	0.54
Wealth	0.03	−0.06	0.13	0.04	−0.06	0.13	0.03	−0.06	0.13
Family size	−0.02	−0.06	0.03	−0.02	−0.07	0.03	−0.02	−0.06	0.03
	OR	95% CI		OR	95% CI		OR	95% CI	
3. Progression to marriage (*n* = 548)									
Father absent <16	1.39^†^	0.98	1.98						
Father absent 0–7				1.24	0.76	2.02			
Father absent 8–15				1.56^†^	0.97	2.52			
Death							1.29	0.82	2.02
Divorce							1.57	0.91	2.70
Wealth	0.72***	0.65	0.80	0.73***	0.65	0.81	0.72***	0.65	0.80
Family size	1.00	0.96	1.05	1.00	0.96	1.05	1.00	0.96	1.05
4. Progression to first birth (*n* = 548)									
Father absent <16	1.50*	1.03	2.18						
Father absent 0–7				1.28	0.76	2.15			
Father absent 8–15				1.77*	1.07	2.91			
Death							1.56^†^	0.96	2.52
Divorce							1.43	0.81	2.52
Wealth	0.73***	0.65	0.81	0.73***	0.66	0.82	0.73***	0.65	0.82
Family size	1.03	0.98	1.08	1.03	0.9′8	1.08	1.03	0.98	1.08
5. Progression from marriage to first birth (*n* = 328)									
Father absent <16	1.21	0.73	2.00						
Father absent 0–7				1.06	0.53	2.13			
Father absent 8–15				1.38	0.70	2.71			
Death							1.83^†^	0.94	3.55
Divorce							0.74	0.36	1.55
Wealth	0.91	0.79	1.04	0.91	0.79	1.04	0.91	0.80	1.04
Family size	1.07*	1.00	1.15	1.08*	1.01	1.15	1.08*	1.01	1.15
6. Progression from first to second birth (*n* = 287)									
Father absent <16	1.31	0.80	2.15						
Father absent 0–7				1.76	0.87	3.53			
Father absent 8–15				1.03	0.53	2.00			
Death							1.51	0.81	2.82
Divorce							1.08	0.51	2.29
Wealth	0.98	0.85	1.13	0.98	0.86	1.13	0.97	0.85	1.12
Family size	0.99	0.93	1.05	0.99	0.93	1.05	0.99	0.93	1.05

All models are adjusted for ethnicity, birth order, birth order squared, the puberty model adjusts for mother’s age at menarche, EHA models also adjust for year of birth, age and age squared

Ref category: father present. *β Coeff.* beta coefficient, *OR* odds ratio, *CI* 95% confidence intervals

*** *p* < 0.001, ** *p* < 0.01, * *p* < 0.05, ^†^ *p* < 0.10

### Age at Menarche

In this population we found no significant association between father absence and the timing of menarche. When we split father absence by cause, we again found no significant effect of either death or marital breakdown. This finding suggests that fathers do not have the same accelerating influence on women’s age at menarche in transitional Malaysia as they do in low fertility settings. Because we only have age at menarche measured in years, it is possible that very small effects of father absence, perhaps by shifting the timing of menarche byonly a few months, would be apparent had we used a more fine-grained variable. However, although the direction of the effect for all-age father absence suggests accelerated pubertal development, the point estimates for each father-absence category are not consistent (some indicate earlier, and some later, puberty), suggesting that there really is little effect of father absence on age at puberty in this sample.

Father-absence effects may be mediated by resource stress, so we included a wealth measure in the models. Contrary to our expectations, we found no significant relationship between parental wealth and age at menarche; nor does including the wealth variable alter the father-absence results. Finally, we included family size in the model and found that larger sibling groups predicted later age at menarche. Besides mother’s age at menarche, family size was the only significant predictor in these models, suggesting that it is a more important influence on age at menarche than father absence in this population.

### Progression to Marriage and First Birth

**Fig. 3:**
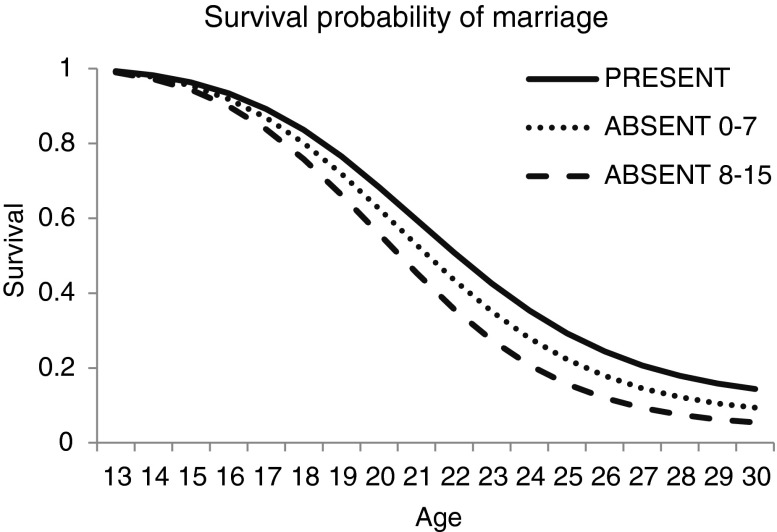
Survival probability of marriage, by age groups of father absence

**Fig. 4:**
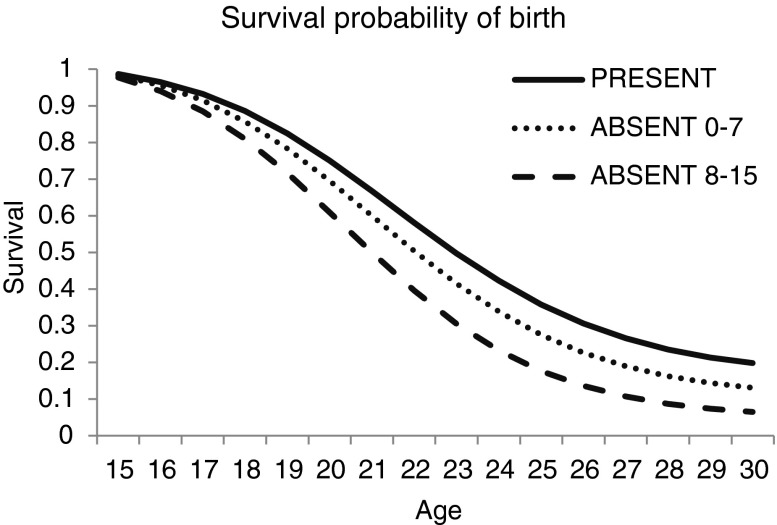
Survival probability of first birth, by age groups of father absence

As expected, there were few differences in the outcomes of the age at marriage and first birth models because these two events are so highly correlated. We found that father absence was significantly associated with faster progression to both marriage and first birth, although the father-absence association with marriage is marginally significant at the 10% level. When we split the father-absence event into early or later childhood we found that, for both outcomes, losing a father at an older age was more important than losing him early (Figs. 3 and 4). The estimated odds of having a first birth in any given year is 1.77 times greater for women who had lost their father during later childhood compared with those whose fathers were always present (see Table 2). For progression to marriage and first birth, the odds ratios suggest faster progressions for women with both dead and divorced fathers, but the only marginally significant outcome is the association with paternal death. Unlike the menarche model, family size is not associated with progression to marriage or first birth, although wealth is. Girls who come from richer parental households tend to have a decreased likelihood of progressing to marriage and first birth per unit time than girls from poorer backgrounds.

### Birth Spacing

**Fig. 5:**
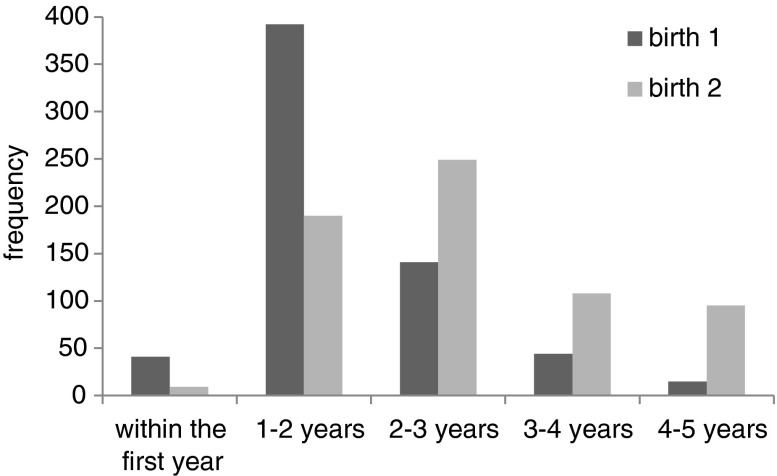
Distribution of time from marriage to first birth and progression to the second birth

Overall, father absence was not significantly associated with progression from marriage to first birth in this population, nor was there any association between early or later father absence and this progression. There was a marginally significant positive association between father’s death and progression to first birth (from marriage): girls whose fathers had died progressed more quickly from marriage to first birth. In contrast, father absence from divorce is associated with *lower* odds of progressing to first birth, though this association was not statistically significant. The different directions of these effects may contribute to the lack of an overall father absence effect. However, there is very little variation in the progression from marriage to first birth, which might explain why there is little association between father absence and this progression. Figure 5 illustrates that 94% of all women have their first child within the first 3 years of marriage (and 65% between years 1 and 2). As shown in Fig. 5, there is a lot more variation in the time between first and second births, although we found no statistically significant associations between any variable in our model, including father absence, and this outcome (see Table 2). Wealth was not correlated with either measure of birth spacing, but family size was negatively associated with the length of the first birth interval: girls from larger families had faster progressions from marriage to first birth.

### Desired Completed Family Size

We found no relationship between father absence and desired family size (see Table 2). There were also no significant relationships found between wealth or family size and desired fertility.

## Discussion

In summary, we found a significant association between father absence during later childhood and more rapid progression to marriage and first birth. In these models, parental wealth was a strong indicator of postponing marriage and children, but father absence was associated with these two outcomes even when wealth was included in the models. We found little effect of father absence on age at puberty, progression from marriage to first birth, or progression to a second birth, nor was there a correlation between father absence and ideal family size. We discuss these findings in more detail below.

### Age at Menarche

In a developing world context, we know little about the relationship between father absence and age at menarche. We do know that resource stress is associated with later ages at menarche, so we might extrapolate that if father absence results in diminished resources, father absence would be associated with delayed menarche. In contrast, in a post–demographic transition setting both father absence and resource stress (measured as low SES) are consistently associated with accelerated pubertal development (although see Campbell and Udry 1995). The reason for this context-dependent outcome is because in high-income countries, where resource stress does not lead to deprived nutrition, father absence is considered a “medium-level” stress, possibly indicative of increased mortality risk, which elicits a faster life history strategy (Ellis 2004).

One way to determine whether resources are a factor in the relationship between father absence and menarche is to include a wealth measure in the models. We did do this, but we found no association between parental wealth and age at menarche. Our measure of wealth is rather crude—it only accounts for the possessions in the parental home at the time of interview, which may not be a good indicator of early life nutrition. However, we did find that large family size, regardless of birth order, was associated with later age at menarche. This might be a better indicator of nutritional stress since siblings will compete for resources within the household. Lack of resources predicting later maturity is characteristic of the low-income pattern, but some studies in high-income settings also report that larger family size promotes later puberty (Bogaert 2008; Coall and Chisholm 2010; Hoier 2003; Padez 2003; but see Tavares et al. 2000), which may argue against the suggestion that large families indicate resource stress. Other studies that examine within-family variation in health outcomes have found that, in the UK, greater numbers of siblings, particularly for later-born children, are associated with stunted growth patterns (Lawson and Mace 2008). This finding is maintained even after controlling for various other aspects of wealth, suggesting that sibling competition for resources is an important factor even in a relatively wealthy setting. Thus, the association between larger sibships and delayed age at puberty in our study could be due to a longer growth period being required in order to reach an adequate stature for sexual maturity to commence. It is also possible that girls delay sexual maturity, especially if there is an absent father, in order to help their mothers to raise their younger siblings (Hoier 2003), although this would mean that lower-birth-order children should be affected—in other words, older sisters are more useful for alloparenting than younger children. To test this we included an interaction term between family size and birth order in the models, but it was non-significant, suggesting that this is not the case in our sample.

Another way to examine whether father absence is an example of resource stress is to include a marker of the daughters’ nutritional status in the model, as this may be a mediating factor. No measures of height or weight were taken at the time of the interviews; however, we do have some information on their birth weight. When we included birth weight as a predictor in the model (results available on request), we found no association with age at menarche, nor did the results change for the father-absence variables. The birth weight data, being retrospectively reported by the mother, could be inaccurate. Furthermore, the sample size is reduced to 96 daughters owing to missing birth-weight information, so our results have little statistical power.

### Marriage and Children

We found a significant association between father absence and more rapid progression to marriage and birth in this sample. These associations were seen over and above other indicators of a stressed early environment (parental wealth and sibship size), suggesting there may be something special about fathers in this population. When we tested whether the timing of father absence made a difference, we found that father absence occurring during later childhood was most important. Here, the explanation may be that father absence puts the household under additional economic stress, which older girls are able to offset by getting married sooner. If fathers become absent during early childhood then the household may be able to recuperate some wealth, particularly if the mother is able to remarry relatively soon. It would be useful to examine whether the length of time a girl is without her father has an influence on age at marriage and first birth. Unfortunately we were unable to do this in the current study owing to the relatively small sample size of girls without fathers, but this could be tested with larger datasets. Furthermore, it would be useful to test whether the introduction of a stepfather into the family makes a difference. Again, though we do have information on whether the mother remarried in this dataset, sample sizes of girls with stepfathers are too small to run robust statistical analyses. It is also difficult to predict the role that stepfathers play: this relationship could increase psychosocial stress for girls (Daly and Wilson 1998), resulting in earlier reproductive maturation, as has been found in two high-income settings (Amato and Kane 2011; Quinlan 2003), or it could have a positive influence if the addition of a stepfather results in improved wealth status for the family.

In terms of the cause of father absence, once adjusted for potentially confounding factors, our models suggest that the death of the father may be somewhat more important than divorce, as there were marginally significant associations between paternal death and progression to first birth (for all women) and progression from marriage to first birth (for married women only) but no significant associations between divorce and any reproductive outcome (although our sample size for divorce is small). This contrasts with industrialized populations, where divorce is often found to be more important. In another transitional population, however, Shenk et al. (2013) found that divorce had an accelerating effect on a rural Bangladeshi woman’s age at marriage and first birth, although the death of a father was associated with delayed marriage and first births. This demonstrates that even among transitioning populations, there are context-dependent effects.

The finding that father absence is not associated with desired family size in our study suggests that some factors affecting earlier reproduction do not predict completed family size. The main assumption here is that desired family size is a good proxy for actual completed fertility, and although some evidence supports this notion (Bankole and Westoff 1998; DeSilva 1991), some does not (Westoff et al. 1957).

Finally, we relate our findings to the four hypotheses that have been proposed to explain the link between fathers and reproduction-related behavior: (1) father absence may be an indicator of a stressed early environment; (2) father absence may be a mortality cue; (3) father absence may indicate the degree to which a woman can expect paternal investment in her future reproductive career; (4) paternal investment may directly influence the reproductive behavior of his offspring. In terms of the overall direction of the correlation between father absence and reproductive timings, our finding that father absence broadly accelerates these events is in line with the predictions of hypotheses 1–3, but cannot speak to hypothesis 4, since this hypothesis doesn’t make a clear prediction about the direction of father-absence effects. However, when we examine timing effects, we find that father absence in later childhood is more important than absence in early childhood, which provides support for hypothesis 4 but not for hypotheses 1–3, all of which rely on father absence during a sensitive period in early childhood. Our finding that father’s death may be more predictive than parental divorce suggests support for hypothesis 2 and 4 (assuming divorced fathers are still providing some investment), but not hypotheses 1 (since father absence through either death or divorce should cause psychosocial stress) or 3 (since parental divorce should be more predictive of reproductive behavior than father’s death). In summary, we suggest our findings are most supportive of the fourth hypothesis, which states that fathers are doing something practical in terms of investment in their children, rather than the first three hypotheses which state that fathers are a cue to some aspect of the child’s environment.

## Limitations

Correlational studies such as these cannot control for all possible confounders and cannot make strong claims about causation. We are also unable to say anything about the quality of paternal relations or other qualitative aspects of paternal investment because we were only able to determine if the mother’s marriage was no longer intact. We did use other proxies to elucidate what father absence means in terms of resources or general stress, but these are only proxies and can be interpreted in more than one way. For example, we use family size as a proxy for resource stress, but we cannot be sure that there isn’t something systematically different about large sibships that drives their association with later menarche. We were also unable to incorporate the availability of alloparents who may mitigate the effects of father absence—for example, if father absence results in extra grandparental effort from the mother’s parents—as we have no data on the availability of such alloparents. Finally, our modest sample size does not allow us to examine interaction effects between wealth and the causes or timing of father absence. Future studies using larger data sets are encouraged to do this in more detail.

## Conclusion

We find that father absence is correlated with faster progression to marriage and first birth in a population undergoing the demographic transition. This is similar to the correlation found in lower-fertility, post-transition societies, but it contrasts with the few studies performed in higher-fertility, pre-transition societies, where father absence is often correlated with delayed, rather than accelerated, reproduction. Our finding that father absence during later childhood is most important is dissimilar to many high-income studies, which tend to find early childhood to be more salient (though this is not a consistent finding even in such settings). Interestingly, our findings also differ from those in low-fertility contexts in that we found no relationship between father absence and age at menarche: in post–demographic transition settings, consistent and significant associations between father absence and earlier menarche persist. Overall, these differences suggest that the mechanisms that bring about accelerated reproduction in father-absent girls may differ between this transitional setting and low-fertility settings; although a better understanding of the pathways through which father absence affects reproductive outcomes in all contexts is needed.

## Electronic supplementary material

Below is the link to the electronic supplementary material.

ESM 1 (DOCX 50.9 kb)
