# Supplementary material for: Father Absence and Reproduction-Related Outcomes in Malaysia, a Transitional Fertility Population
Source: Hum Nat. 2014 Mar 8;25(2):213–34. doi: 10.1007/s12110-014-9195-2 (PMC4052008; doi:10.1007/s12110-014-9195-2)
Supplement: Supplementary file 1 — (DOCX 50.9 kb) [file 12110_2014_9195_MOESM1_ESM.docx]

ESM for Father Absence and Reproduction-Related Outcomes in Malaysia, a Transitional Fertility Population, by Paula Sheppard, Kristin Snopkowski, Rebecca Sear

Published in 2014 in *Human Nature* 25(2), doi: [10.1007/s12110-014-9195-2](http://dx.doi.org/10.1007/s12110-014-9195-2)

| LINEAR REGRESSION MODEL |  | |  | |  | |  | |  | |  | |  | |
| --- | --- | --- | --- | --- | --- | --- | --- | --- | --- | --- | --- | --- | --- | --- |
| **age at menarche** | **Coef.** | | **S.E.** | | **t** | | **P>t** | | **95% C.I.** | | | | **n=249** | |
| father absent at age 15 | -0.30 | | 0.28 | | -1.06 | | 0.288 | | -0.85 | | 0.25 | |  | |
| mother's age at menarche | 0.24 | | 0.05 | | 4.88 | | 0.000 | | 0.14 | | 0.34 | |  | |
| maternal household wealth | -0.07 | | 0.07 | | -1.07 | | 0.286 | | -0.21 | | 0.06 | |  | |
| Malay | 0.30 | | 0.19 | | 1.53 | | 0.128 | | -0.09 | | 0.68 | |  | |
| family size | 0.10 | | 0.03 | | 3.07 | | 0.002 | | 0.04 | | 0.17 | |  | |
| birth order | 0.03 | | 0.15 | | 0.21 | | 0.835 | | -0.26 | | 0.32 | |  | |
| birth order² | 0.00 | | 0.02 | | -0.15 | | 0.878 | | -0.04 | | 0.03 | |  | |
| intercept | 9.39 | | 0.80 | | 11.74 | | 0.000 | | 7.82 | | 10.97 | |  | |
| **age at menarche** | **Coef.** | | **S.E.** | | **t** | | **P>t** | | **95% C.I.** | | | | **n=259** | |
| father absent birth to 7 | -0.56 | | 0.35 | | -1.58 | | 0.114 | | -1.25 | | 0.14 | |  | |
| father absent 8 to 15 | 0.12 | | 0.44 | | 0.26 | | 0.794 | | -0.76 | | 0.99 | |  | |
| mother's age at menarche | 0.23 | | 0.05 | | 4.72 | | 0.000 | | 0.14 | | 0.33 | |  | |
| maternal household wealth | -0.07 | | 0.07 | | -1.04 | | 0.300 | | -0.21 | | 0.06 | |  | |
| Malay | 0.30 | | 0.19 | | 1.55 | | 0.123 | | -0.08 | | 0.68 | |  | |
| family size | 0.10 | | 0.03 | | 3.04 | | 0.003 | | 0.04 | | 0.17 | |  | |
| birth order | 0.02 | | 0.15 | | 0.11 | | 0.915 | | -0.27 | | 0.30 | |  | |
| birth order² | 0.00 | | 0.02 | | -0.10 | | 0.922 | | -0.03 | | 0.03 | |  | |
| intercept | 9.51 | | 0.81 | | 11.81 | | 0.000 | | 7.93 | | 11.10 | |  | |
| **age at menarche** | **Coef.** | | **S.E.** | | **t** | | **P>t** | | **95% C.I.** | | | | **n=249** | |
| death | 0.02 | | 0.39 | | 0.06 | | 0.950 | | -0.74 | | 0.79 | |  | |
| divorce | -0.63 | | 0.39 | | -1.60 | | 0.111 | | -1.40 | | 0.14 | |  | |
| mother's age at menarche | 0.24 | | 0.05 | | 4.91 | | 0.000 | | 0.14 | | 0.34 | |  | |
| maternal household wealth | -0.08 | | 0.07 | | -1.08 | | 0.280 | | -0.21 | | 0.06 | |  | |
| Malay | 0.34 | | 0.20 | | 1.73 | | 0.085 | | -0.05 | | 0.73 | |  | |
| family size | 0.11 | | 0.03 | | 3.11 | | 0.002 | | 0.04 | | 0.17 | |  | |
| birth order | 0.02 | | 0.15 | | 0.11 | | 0.914 | | -0.27 | | 0.30 | |  | |
| birth order² | 0.00 | | 0.02 | | -0.11 | | 0.910 | | -0.04 | | 0.03 | |  | |
| intercept | 9.36 | | 0.80 | | 11.71 | | 0.000 | | 7.79 | | 10.94 | |  | |
| POISSON MODEL: |  | |  | |  | |  | |  | |  | |  | |
| **ideal family size** | **Coef.** | | **S.E.** | | **t** | | **P>t** | | **95% C.I.** | | | | **n=141** | |
| father absent at age 15 | 0.05 | | 0.18 | | 0.27 | | 0.783 | | -0.30 | | 0.40 | |  | |
| maternal household wealth | 0.03 | | 0.05 | | 0.70 | | 0.483 | | -0.06 | | 0.13 | |  | |
| Malay | 0.50 | | 0.15 | | 3.22 | | 0.001 | | 0.19 | | 0.80 | |  | |
| family size | -0.02 | | 0.02 | | -0.80 | | 0.422 | | -0.06 | | 0.03 | |  | |
| birth order | 0.00 | | 0.09 | | -0.04 | | 0.966 | | -0.18 | | 0.17 | |  | |
| birth order² | 0.01 | | 0.01 | | 0.54 | | 0.588 | | -0.01 | | 0.02 | |  | |
| intercept | 0.43 | | 0.29 | | 1.45 | | 0.146 | | -0.15 | | 1.01 | |  | |
| **ideal family size** | **Coef.** | | **S.E.** | | **t** | | **P>t** | | **95% C.I.** | | | | **n=141** | |
| father absent birth to 7 | -0.01 | | 0.24 | | -0.05 | | 0.958 | | -0.48 | | 0.45 | |  | |
| father absent 8 to 15 | 0.13 | | 0.26 | | 0.50 | | 0.616 | | -0.39 | | 0.65 | |  | |
| maternal household wealth | 0.04 | | 0.05 | | 0.75 | | 0.453 | | -0.06 | | 0.13 | |  | |
| Malay | 0.50 | | 0.15 | | 3.23 | | 0.001 | | 0.20 | | 0.80 | |  | |
| family size | -0.02 | | 0.02 | | -0.83 | | 0.408 | | -0.07 | | 0.03 | |  | |
| birth order | -0.01 | | 0.09 | | -0.09 | | 0.930 | | -0.18 | | 0.17 | |  | |
| birth order² | 0.01 | | 0.01 | | 0.57 | | 0.567 | | -0.01 | | 0.02 | |  | |
| intercept | 0.43 | | 0.30 | | 1.46 | | 0.143 | | -0.15 | | 1.01 | |  | |
| **ideal family size** | | **Coef.** | | **S.E.** | | **t** | | **P>t** | | **95% C.I.** | | | | **n=141** |
| death | | 0.04 | | 0.25 | | 0.17 | | 0.865 | | -0.45 | | 0.54 | |  |
| divorce | | 0.06 | | 0.25 | | 0.23 | | 0.819 | | -0.43 | | 0.54 | |  |
| maternal household wealth | | 0.03 | | 0.05 | | 0.70 | | 0.483 | | -0.06 | | 0.13 | |  |
| Malay | | 0.50 | | 0.16 | | 3.20 | | 0.001 | | 0.19 | | 0.80 | |  |
| family size | | -0.02 | | 0.02 | | -0.80 | | 0.422 | | -0.06 | | 0.03 | |  |
| birth order | | 0.00 | | 0.09 | | -0.04 | | 0.969 | | -0.18 | | 0.17 | |  |
| birth order² | | 0.01 | | 0.01 | | 0.54 | | 0.589 | | -0.01 | | 0.02 | |  |
| intercept | | 0.43 | | 0.29 | | 1.45 | | 0.146 | | -0.15 | | 1.01 | |  |
| DISCRETE-TIME EVENT-HISTORY MODELS: | |  | |  | |  | |  | |  | |  | |  |
| **progression to marriage** | | **O. R.** | | **S.E.** | | **z** | | **P>z** | | **95% C.I.** | | | | **n=548** |
| father absent at age 15 | | 1.39 | | 0.25 | | 1.83 | | 0.067 | | 0.98 | | 1.98 | |  |
| maternal household wealth | | 0.72 | | 0.04 | | -6.05 | | 0.000 | | 0.65 | | 0.80 | |  |
| Malay | | 1.28 | | 0.17 | | 1.84 | | 0.066 | | 0.98 | | 1.67 | |  |
| family size | | 1.00 | | 0.02 | | 0.18 | | 0.858 | | 0.96 | | 1.05 | |  |
| birth order | | 1.02 | | 0.08 | | 0.23 | | 0.822 | | 0.87 | | 1.20 | |  |
| birth order² | | 1.00 | | 0.01 | | -0.32 | | 0.750 | | 0.98 | | 1.01 | |  |
| period | | 3.49 | | 0.56 | | 7.71 | | 0.000 | | 2.54 | | 4.79 | |  |
| period² | | 0.98 | | 0.00 | | -6.47 | | 0.000 | | 0.97 | | 0.98 | |  |
| year born | | 0.93 | | 0.01 | | -6.11 | | 0.000 | | 0.91 | | 0.95 | |  |
| intercept | | 0.00 | | 0.00 | | -6.80 | | 0.000 | | 0.00 | | 0.00 | |  |
| **progression to marriage** | | **O. R.** | | **S.E.** | | **z** | | **P>z** | | **95% C.I.** | | | | **n-548** |
| father absent birth to 7 | | 1.24 | | 0.31 | | 0.87 | | 0.383 | | 0.76 | | 2.02 | |  |
| father absent 8 to 15 | | 1.56 | | 0.38 | | 1.84 | | 0.066 | | 0.97 | | 2.52 | |  |
| maternal household wealth | | 0.73 | | 0.04 | | -6.00 | | 0.000 | | 0.65 | | 0.81 | |  |
| Malay | | 1.29 | | 0.18 | | 1.89 | | 0.059 | | 0.99 | | 1.69 | |  |
| family size | | 1.00 | | 0.02 | | 0.22 | | 0.829 | | 0.96 | | 1.05 | |  |
| birth order | | 1.01 | | 0.08 | | 0.16 | | 0.871 | | 0.86 | | 1.19 | |  |
| birth order² | | 1.00 | | 0.01 | | -0.26 | | 0.791 | | 0.98 | | 1.01 | |  |
| period | | 3.49 | | 0.56 | | 7.71 | | 0.000 | | 2.54 | | 4.79 | |  |
| period² | | 0.97 | | 0.00 | | -6.47 | | 0.000 | | 0.97 | | 0.98 | |  |
| year born | | 0.93 | | 0.01 | | -6.13 | | 0.000 | | 0.91 | | 0.95 | |  |
| intercept | | 0.00 | | 0.00 | | -6.80 | | 0.000 | | 0.00 | | 0.00 | |  |
| **progression to marriage** | | **O. R.** | | **S.E.** | | **z** | | **P>z** | | **95% C.I.** | | | | **n=548** |
| death | | 1.29 | | 0.30 | | 1.10 | | 0.271 | | 0.82 | | 2.02 | |  |
| divorce | | 1.57 | | 0.43 | | 1.61 | | 0.106 | | 0.91 | | 2.70 | |  |
| maternal household wealth | | 0.72 | | 0.04 | | -6.07 | | 0.000 | | 0.65 | | 0.80 | |  |
| Malay | | 1.26 | | 0.18 | | 1.68 | | 0.092 | | 0.96 | | 1.66 | |  |
| family size | | 1.00 | | 0.02 | | 0.16 | | 0.870 | | 0.96 | | 1.05 | |  |
| birth order | | 1.02 | | 0.08 | | 0.27 | | 0.790 | | 0.87 | | 1.20 | |  |
| birth order² | | 1.00 | | 0.01 | | -0.33 | | 0.742 | | 0.98 | | 1.01 | |  |
| period | | 3.50 | | 0.57 | | 7.72 | | 0.000 | | 2.55 | | 4.81 | |  |
| period² | | 0.97 | | 0.00 | | -6.49 | | 0.000 | | 0.97 | | 0.98 | |  |
| year born | | 0.93 | | 0.01 | | -6.02 | | 0.000 | | 0.91 | | 0.95 | |  |
| intercept | | 0.00 | | 0.00 | | -6.82 | | 0.000 | | 0.00 | | 0.00 | |  |

| **progression to first birth** | **O. R.** | **S.E.** | **z** | **P>z** | **95% C.I.** | | **n=548** |
| --- | --- | --- | --- | --- | --- | --- | --- |
| father absent at age 15 | 1.50 | 0.29 | 2.13 | 0.033 | 1.03 | 2.18 |  |
| maternal household wealth | 0.73 | 0.04 | -5.61 | 0.000 | 0.65 | 0.81 |  |
| Malay | 1.36 | 0.20 | 2.11 | 0.035 | 1.02 | 1.80 |  |
| family size | 1.03 | 0.02 | 1.04 | 0.299 | 0.98 | 1.08 |  |
| birth order | 1.01 | 0.10 | 0.14 | 0.887 | 0.84 | 1.22 |  |
| birth order² | 0.99 | 0.01 | -0.78 | 0.437 | 0.97 | 1.01 |  |
| period | 4.05 | 0.84 | 6.75 | 0.000 | 2.70 | 6.08 |  |
| period² | 0.97 | 0.00 | -5.94 | 0.000 | 0.96 | 0.98 |  |
| year born | 0.94 | 0.01 | -4.94 | 0.000 | 0.92 | 0.96 |  |
| intercept | 0.00 | 0.00 | -6.42 | 0.000 | 0.00 | 0.00 |  |
| **progression to first birth** | **O. R.** | **S.E.** | **z** | **P>z** | **95% C.I.** | | **n=548** |
| father absent birth to 7 | 1.28 | 0.34 | 0.92 | 0.357 | 0.76 | 2.15 |  |
| father absent 8 to 15 | 1.77 | 0.45 | 2.24 | 0.025 | 1.07 | 2.91 |  |
| maternal household wealth | 0.73 | 0.04 | -5.55 | 0.000 | 0.66 | 0.82 |  |
| Malay | 1.38 | 0.20 | 2.19 | 0.028 | 1.03 | 1.83 |  |
| family size | 1.03 | 0.03 | 1.08 | 0.281 | 0.98 | 1.08 |  |
| birth order | 1.01 | 0.09 | 0.07 | 0.946 | 0.84 | 1.21 |  |
| birth order² | 0.99 | 0.01 | -0.72 | 0.471 | 0.97 | 1.01 |  |
| period | 4.04 | 0.84 | 6.74 | 0.000 | 2.69 | 6.07 |  |
| period² | 0.97 | 0.00 | -5.93 | 0.000 | 0.96 | 0.98 |  |
| year born | 0.94 | 0.01 | -4.96 | 0.000 | 0.92 | 0.96 |  |
| intercept | 0.00 | 0.00 | -6.41 | 0.000 | 0.00 | 0.00 |  |
| **progression to first birth** | **O. R.** | **S.E.** | **z** | **P>z** | **95% C.I.** | | **n=548** |
| death | 1.56 | 0.38 | 1.81 | 0.071 | 0.96 | 2.52 |  |
| divorce | 1.43 | 0.41 | 1.22 | 0.222 | 0.81 | 2.52 |  |
| maternal household wealth | 0.73 | 0.04 | -5.59 | 0.000 | 0.65 | 0.82 |  |
| Malay | 1.37 | 0.20 | 2.11 | 0.035 | 1.02 | 1.83 |  |
| family size | 1.03 | 0.02 | 1.04 | 0.298 | 0.98 | 1.08 |  |
| birth order | 1.01 | 0.10 | 0.13 | 0.900 | 0.84 | 1.22 |  |
| birth order² | 0.99 | 0.01 | -0.77 | 0.439 | 0.97 | 1.01 |  |
| period | 4.04 | 0.84 | 6.73 | 0.000 | 2.69 | 6.07 |  |
| period² | 0.97 | 0.00 | -5.92 | 0.000 | 0.96 | 0.98 |  |
| year born | 0.94 | 0.01 | -4.93 | 0.000 | 0.92 | 0.96 |  |
| intercept | 0.00 | 0.00 | -6.38 | 0.000 | 0.00 | 0.00 |  |
| **progression from marriage to first birth** | **O. R.** | **S.E.** | **z** | **P>z** | **95% C.I.** | | **n=328** |
| father absent at age 15 | 1.21 | 0.31 | 0.75 | 0.453 | 0.73 | 2.00 |  |
| maternal household wealth | 0.91 | 0.06 | -1.40 | 0.163 | 0.79 | 1.04 |  |
| Malay | 1.11 | 0.21 | 0.57 | 0.569 | 0.77 | 1.61 |  |
| family size | 1.07 | 0.04 | 2.10 | 0.036 | 1.00 | 1.15 |  |
| birth order | 0.99 | 0.13 | -0.05 | 0.962 | 0.77 | 1.29 |  |
| birth order² | 0.99 | 0.01 | -1.08 | 0.279 | 0.96 | 1.01 |  |
| period | 165.52 | 73.28 | 11.54 | 0.000 | 69.50 | 394.16 |  |
| period² | 0.37 | 0.03 | -10.53 | 0.000 | 0.31 | 0.45 |  |
| year born | 0.98 | 0.02 | -1.21 | 0.225 | 0.95 | 1.01 |  |
| intercept | 0.01 | 0.01 | -4.47 | 0.000 | 0.00 | 0.06 |  |

| **progression from marriage to first birth** | **O. R.** | **S.E.** | **z** | **P>z** | **95% C.I.** | | **n=328** |
| --- | --- | --- | --- | --- | --- | --- | --- |
| father absent birth to 7 | 1.06 | 0.38 | 0.16 | 0.872 | 0.53 | 2.13 |  |
| father absent 8 to 15 | 1.38 | 0.47 | 0.93 | 0.352 | 0.70 | 2.71 |  |
| maternal household wealth | 0.91 | 0.06 | -1.36 | 0.175 | 0.79 | 1.04 |  |
| Malay | 1.13 | 0.21 | 0.62 | 0.533 | 0.78 | 1.63 |  |
| family size | 1.08 | 0.04 | 2.12 | 0.034 | 1.01 | 1.15 |  |
| birth order | 0.99 | 0.13 | -0.11 | 0.913 | 0.76 | 1.28 |  |
| birth order² | 0.99 | 0.01 | -1.04 | 0.298 | 0.96 | 1.01 |  |
| period | 165.09 | 73.10 | 11.53 | 0.000 | 69.31 | 393.22 |  |
| period² | 0.37 | 0.03 | -10.52 | 0.000 | 0.31 | 0.45 |  |
| year born | 0.98 | 0.02 | -1.26 | 0.208 | 0.95 | 1.01 |  |
| intercept | 0.01 | 0.01 | -4.41 | 0.000 | 0.00 | 0.07 |  |
| **progression from marriage to first birth** | **O. R.** | **S.E.** | **z** | **P>z** | **95% C.I.** | | **n=328** |
| Death | 1.83 | 0.62 | 1.78 | 0.075 | 0.94 | 3.55 |  |
| divorce | 0.74 | 0.28 | -0.79 | 0.431 | 0.36 | 1.55 |  |
| maternal household wealth | 0.90 | 0.06 | -1.45 | 0.147 | 0.78 | 1.04 |  |
| Malay | 1.20 | 0.23 | 0.93 | 0.352 | 0.82 | 1.75 |  |
| family size | 1.08 | 0.04 | 2.12 | 0.034 | 1.01 | 1.15 |  |
| birth order | 0.98 | 0.13 | -0.13 | 0.893 | 0.76 | 1.27 |  |
| birth order² | 0.99 | 0.01 | -1.08 | 0.280 | 0.96 | 1.01 |  |
| period | 168.59 | 74.81 | 11.55 | 0.000 | 70.65 | 402.30 |  |
| period² | 0.37 | 0.03 | -10.53 | 0.000 | 0.31 | 0.45 |  |
| year born | 0.98 | 0.02 | -1.47 | 0.143 | 0.94 | 1.01 |  |
| intercept | 0.01 | 0.01 | -4.23 | 0.000 | 0.00 | 0.08 |  |
| **progression from first to second birth** | **O. R.** | **S.E.** | **z** | **P>z** | **95% C.I.** | | **n-=287** |
| father absent at age 15 | 1.31 | 0.33 | 1.07 | 0.284 | 0.80 | 2.15 |  |
| maternal household wealth | 0.98 | 0.07 | -0.28 | 0.778 | 0.85 | 1.13 |  |
| Malay | 1.02 | 0.19 | 0.12 | 0.904 | 0.71 | 1.48 |  |
| family size | 0.99 | 0.03 | -0.30 | 0.761 | 0.93 | 1.05 |  |
| birth order | 0.93 | 0.11 | -0.63 | 0.532 | 0.74 | 1.17 |  |
| birth order² | 1.01 | 0.01 | 0.55 | 0.581 | 0.98 | 1.03 |  |
| period | 28.63 | 10.68 | 9.00 | 0.000 | 13.79 | 59.47 |  |
| period² | 0.60 | 0.04 | -8.22 | 0.000 | 0.53 | 0.68 |  |
| year born | 0.95 | 0.01 | -3.11 | 0.002 | 0.92 | 0.98 |  |
| intercept | 0.07 | 0.07 | -2.58 | 0.010 | 0.01 | 0.52 |  |
| **progression from first to second birth** | **O. R.** | **S.E.** | **z** | **P>z** | **95% C.I.** | | **n=287** |
| father absent birth to 7 | 1.76 | 0.63 | 1.58 | 0.115 | 0.87 | 3.53 |  |
| father absent 8 to 15 | 1.03 | 0.35 | 0.08 | 0.934 | 0.53 | 2.00 |  |
| maternal household wealth | 0.98 | 0.07 | -0.25 | 0.803 | 0.86 | 1.13 |  |
| Malay | 1.02 | 0.19 | 0.12 | 0.901 | 0.71 | 1.48 |  |
| family size | 0.99 | 0.03 | -0.41 | 0.684 | 0.93 | 1.05 |  |
| birth order | 0.95 | 0.11 | -0.47 | 0.638 | 0.75 | 1.19 |  |
| birth order² | 1.01 | 0.01 | 0.49 | 0.624 | 0.98 | 1.03 |  |
| period | 28.88 | 10.78 | 9.01 | 0.000 | 13.89 | 60.04 |  |
| period² | 0.60 | 0.04 | -8.23 | 0.000 | 0.53 | 0.68 |  |
| year born | 0.95 | 0.01 | -3.03 | 0.002 | 0.93 | 0.98 |  |
| intercept | 0.06 | 0.06 | -2.66 | 0.008 | 0.01 | 0.48 |  |

| **progression from first to second birth** | **O. R.** | **S.E.** | **z** | **P>z** | **95% C.I.** | | **n=287** |
| --- | --- | --- | --- | --- | --- | --- | --- |
| death | 1.51 | 0.48 | 1.28 | 0.200 | 0.81 | 2.82 |  |
| divorce | 1.08 | 0.41 | 0.19 | 0.850 | 0.51 | 2.29 |  |
| maternal household wealth | 0.97 | 0.07 | -0.39 | 0.696 | 0.85 | 1.12 |  |
| Malay | 1.04 | 0.20 | 0.21 | 0.837 | 0.72 | 1.51 |  |
| family size | 0.99 | 0.03 | -0.28 | 0.779 | 0.93 | 1.05 |  |
| birth order | 0.93 | 0.11 | -0.66 | 0.511 | 0.74 | 1.16 |  |
| birth order² | 1.01 | 0.01 | 0.56 | 0.576 | 0.98 | 1.03 |  |
| period | 28.60 | 10.67 | 8.99 | 0.000 | 13.77 | 59.41 |  |
| period² | 0.60 | 0.04 | -8.22 | 0.000 | 0.53 | 0.68 |  |
| year born | 0.95 | 0.01 | -3.17 | 0.002 | 0.92 | 0.98 |  |
| intercept | 0.08 | 0.08 | -2.43 | 0.015 | 0.01 | 0.61 |  |
